# Open versus Robot-Assisted Radical Cystectomy for the Treatment of pT4a Bladder Cancer: Comparison of Perioperative Outcomes

**DOI:** 10.3390/cancers16071329

**Published:** 2024-03-28

**Authors:** Davide Perri, Bernardo Rocco, Maria Chiara Sighinolfi, Pierluigi Bove, Antonio L. Pastore, Alessandro Volpe, Andrea Minervini, Alessandro Antonelli, Stefano Zaramella, Antonio Galfano, Giovanni E. Cacciamani, Antonio Celia, Orietta Dalpiaz, Simone Crivellaro, Francesco Greco, Giovannalberto Pini, Angelo Porreca, Andrea Pacchetti, Tommaso Calcagnile, Lorenzo Berti, Carlo Buizza, Federica Mazzoleni, Giorgio Bozzini

**Affiliations:** 1 Department of Urology, ASST Lariana, 22100 Como, Italygioboz@yahoo.it (G.B.); 2 Department of Urology, ASST Santi Paolo e Carlo, 20142 Milan, Italy; 3 Department of Urology, Ospedale San Carlo di Nancy, 00165 Rome, Italy; 4 Department of Urology, Sapienza University, 00185 Rome, Italy; 5 Department of Urology, Ospedale Maggiore della Carità, 28100 Novara, Italy; 6 Department of Urology, Azienda Ospedaliero Universitaria Careggi, 50134 Florence, Italy; 7 Department of Urology, Ospedale Maggiore Borgo Trento, 37124 Verona, Italy; 8 Department of Urology, Ospedale degli Infermi, 13875 Biella, Italy; 9 Department of Urology, ASST Grande Ospedale Metropolitano Niguarda, 20162 Milan, Italy; 10 Department of Urology, USC Institute of Urology, Los Angeles, CA 90017, USA; 11 Department of Urology, Ospedale San Bassiano, 36061 Bassano del Grappa, Italy; 12 Department of Urology, Medical University of Graz, 8010 Graz, Austria; 13 Department of Urology, University of Illinois at Chicago, Chicago, IL 60612, USA; 14 Department of Urology, Humanitas Gavazzeni, 24125 Bergamo, Italy; 15 Department of Urology, San Raffaele Turro, 20132 Milan, Italy; 16 Department of Urology, Policlinico Abano Terme, Abano Terme, 35031 Padova, Italy; 17 Department of Urology, Ospedale di Busto Arsizio, 21052 Busto Arsizio, Italy

**Keywords:** bladder cancer, pT4, open radical cystectomy, robot-assisted radical cystectomy

## Abstract

**Simple Summary:**

Bladder cancer is the second most common genitourinary malignancy. Robotic-assisted radical cystectomy has demonstrated comparable efficacy in treating bladder cancer to open radical cystectomy. Due to the features of the procedure itself and the often weak general health status of patients, radical cystectomy is related to a significant incidence of complications. During the last decades, robotic surgery has spread in bladder cancer treatment in order to take advantage of the benefits of minimally invasive surgery. However, the majority of evidence in the literature comes from cT2-T3 muscle-invasive bladder cancer. The management of patients with cT4 stage represents a relevant surgical challenge. The aim of the present study is to compare intra- and postoperative outcomes of robot-assisted and open radical cystectomy in the treatment of patients with a pT4a MIBC in a pathological report.

**Abstract:**

We compared the perioperative outcomes of open (ORC) vs. robot-assisted (RARC) radical cystectomy in the treatment of pT4a MIBC. In total, 212 patients underwent ORC (102 patients, Group A) vs. RARC (110 patients, Group B) for pT4a bladder cancer. Patients were prospectively followed and retrospectively reviewed. We assessed operative time, estimated blood loss (EBL), intraoperative and postoperative complications, length of stay, transfusion rate, and oncological outcomes. Preoperative features were comparable. The mean operative time was 232.8 vs. 189.2 min (*p* = 0.04), and mean EBL was 832.8 vs. 523.7 mL in Group A vs. B (*p* = 0.04). An intraoperative transfusion was performed in 32 (31.4%) vs. 11 (10.0%) cases during ORC vs. RARC (*p* = 0.03). The intraoperative complications rate was comparable. The mean length of stay was shorter after RARC (12.6 vs. 7.2 days, *p* = 0.02). Postoperative transfusions were performed in 36 (35.3%) vs. 13 (11.8%) cases (*p* = 0.03), and postoperative complications occurred in 37 (36.3%) vs. 29 (26.4%) patients in Groups A vs. B (*p* = 0.05). The positive surgical margin (PSM) rate was lower after RARC. No differences were recorded according to the oncological outcomes. ORC and RARC are feasible treatments for the management of pT4a bladder tumors. Minimally invasive surgery provides shorter operative time, bleeding, transfusion rate, postoperative complications, length of stay, and PSM rate.

## 1. Introduction

Bladder cancer is the second most common genitourinary malignancy, with a worldwide age-standardized incidence rate of 9.5 per 100,000 person/years and a mortality rate of 3.3 per 100,000 person/years among men, which are approximately four times higher than those among women globally [1]. More than 85% of patients with muscle-invasive bladder cancer (MIBC), if left untreated, die within 5 years from their diagnosis [2]. Radical cystectomy with pelvic lymph node dissection is considered the standard surgical procedure for patients with very-high-risk non-muscle-invasive bladder cancer (NMIBC) or MIBC staged cT2 to cT4a [3]. For cT4b stages, the guidelines are limited as there are few clinical trials available, due to the high mortality rate and lower incidence [4].

Robotic-assisted radical cystectomy (RARC) has demonstrated comparable efficacy in treating bladder cancer to open radical cystectomy (ORC), with a similar rate of progression-free survival [5]. Due to the advantages of robotic surgery, such as 3D visualization, magnification, and enhanced precision of movements, RARC appears to confer some benefits, including reduced blood loss, lower transfusion rate, faster recovery, and fewer postoperative complications [6,7,8]. Moreover, minimally invasive techniques in general seem to enhance patients’ tolerance and acceptance towards the procedure [9]. From 2004 to 2012, the number of RARCs increased 30-fold, from 0.6% to 18.5% [10,11]. At the beginning, most surgeons were used to performing extracorporeal urinary diversion (ECUD), but intracorporeal urinary diversion (ICUD) has spread, especially in high-volume centers [12].

Several findings from single-institution, multicenter studies, randomized trials, and meta-analyses suggest that RARC has similar long-term oncologic outcomes when compared to ORC. Sathianathen et al. performed a meta-analysis from five randomized controlled trials comparing RARC with ORC. The authors concluded that surgical approach did not have a significant impact on the oncologic outcomes, safety and quality of life of the patients, and that the benefits of robotics were a decreased need for blood transfusion and earlier hospital discharge [13]. The randomized open vs. robotic cystectomy (RAZOR) trial showed no difference in recurrence rate, 3-year progression-free survival, or 3-year overall survival [14]. Data from the International Robotic Cystectomy Consortium suggested that long-term oncologic outcomes after RARC seem comparable to those in open series [15]. In a retrospective single-institution review, higher positive surgical margin rates were observed in the ORC cohort [16].

Due to the extensive surgical burden and the multiple co-morbidities which often affect patients, radical cystectomy is associated with high rates of perioperative complications [17]. Metabolic, infectious, genitourinary, and gastrointestinal complications are the most common causes of readmission after radical cystectomy [18]. The rate of the perioperative complications of RARC reported in the literature should be carefully evaluated, taking into account the learning curve of the procedure, especially when an intracorporeal urinary diversion is performed. Indeed, Clavien–Dindo grade 3–5 complications after RARC with intracorporeal diversion decreased significantly from 25% in 2005 to 6% in 2015, together with the improvement in surgical skills [19]. In a randomized trial comparing RARC to ORC at nine sites in the UK, Catto et al. reported that thromboembolic and wound complications were less common with robotic surgery [7]. Mortezavi et al. reported the results of a cohort study from the Swedish National Register of Urinary Bladder. Compared to ORC, RARC was associated with lower estimated blood loss, a lower intraoperative transfusion rate, a shorter length of stay, and a lower rehospitalization rate. Overall, the RARC group had a lower risk of Clavien–Dindo grade III or higher complications [20]. Similarly, Zhou et al. showed that RARC had lower estimated blood loss, a lower transfusion rate, and a lower rate of high-grade postoperative complications compared to ORC, but longer operative time. Moreover, there was no significant difference in the rate of postoperative low-grade complications [21]. The RACE prospective multicenter comparative effectiveness study showed no statistically significant differences between ORC and RARC in terms of complications and quality of life. Specifically, at 90 postoperative days, the any-grade complication rate was 63% vs. 56%, whereas the major complication rate was 15% vs. 16% after ORC vs. RARC, respectively [22].

Differently from the comparison of the oncological and perioperative outcomes, randomized data on the implications of ICUD vs. ECUD RARC are lacking. Smaller incisions, less pain, faster recovery of bowel function, and reduced risk of fluid imbalance may be obtained from ICUD due to the entirely minimally invasive procedure [23,24]. The use of the intracorporeal approach has increased, especially in high-volume centers, improving outcomes while improving surgical skills [25]. A cohort study showed that ICUD was associated with shorter operative time and less blood loss. ICUD was also associated with more overall complications and readmissions, but not high-grade complications [15]. Another prospective comparison of these two approaches performed by two surgeons showed no differences in postoperative complications either overall or majorly [26]. Interestingly, highly comorbid patients who underwent ICUD had a lower risk of postoperative complications compared to patients who received ECUD, probably as a consequence of less surgical stress, less blood loss, and less need for transfusions or reduced bowel manipulation [27]. In a meta-analysis by Tanneru et al., ICUD and ECUD had a comparable early (30-day) and mid-term (90-day) complication rate. Moreover, high-volume centers had decreased operative times for ICUD [28].

However, the majority of evidence in the literature comes from cT2-T3 MIBC. The management of patients with cT4 stage bladder cancer represents a relevant surgical challenge [29], as locally advanced cT4 tumors may be unresectable, and indication to radical cystectomy is often given with a palliative intent [30]. Considering the potential surgical complexity of cT4 bladder tumors, surgeons sometimes prefer to proceed with an open approach for the fear of intraoperative complications or believing to have a higher rate of oncological radicality. However, there is yet no demonstration of that, and the data in the literature regarding the role of RARC in locally advanced bladder cancer are still limited. The aim of the present study is to compare the intra- and postoperative outcomes of robot-assisted and open radical cystectomy in the treatment of patients with a pT4a MIBC in a pathological report.

## 2. Materials and Methods

Patients submitted to radical cystectomy from 2013 to 2023 for muscle-invasive cancer and whose final pathology revealed a pT4a tumor were considered. Surgery was performed with either an open (Group A) or a robot-assisted (Group B) approach. Twelve urologic departments were involved. In all cases, surgical indication was given following a previous transurethral resection of the bladder (TURB). Preoperative thoraco-abdominal computed tomography was performed in all the patients, and no metastases were found. All surgeries were carried out by expert urologists, and in all cases, RARC was performed with the Xi Da Vinci Robot (Intuitive). In male patients, the prostate and the seminal vesicles were removed together with the bladder. In female patients, the bladder was removed together with the uterus, anterior wall of the vagina, and ovaries in all cases.

Patients were prospectively followed and retrospectively reviewed. We assessed intraoperative and postoperative parameters, specifically operative time, estimated blood loss (EBL), intraoperative and postoperative complications, length of stay, and transfusion rate. Follow-up was performed through regular CT scans, starting three months after surgery and every six months thereafter until 5 years was reached. After that, a CT scan once per year was indicated according to the patient’s general health status. However, a standardized protocol was not defined.

Mean and standard deviation (SD) vs. numbers and proportions were used to describe continuous and categorical variables, respectively. Student’s *t*-test was used to test continuous variables conforming to normal distribution. The chi-square test was used for the comparison of the two study groups. Data were analyzed with R software for statistical computing and graphics version 3.4.1 (R Foundation for Statistical Computing, Vienna, Austria). All statistical tests were two-sided, with a level of significance set at *p* < 0.05.

## 3. Results

Overall, 212 patients were evaluated. Specifically, 102 patients (48.1%) underwent ORC (Group A), and 110 patients (51.9%) underwent RARC (Group B). The descriptive characteristics of the cohorts are summarized in Table 1. Mean age was 78.9 years in Group A and 79.2 years in Group B (*p* = 0.19). Patients undergoing ORC had a mean BMI of 25.8 kg/m^2^, mean age-adjusted Charlson Comorbidity Index (CCI) of 6.9, and a mean American Society of Anesthesiologists (ASAs) score of 2.9. Patients undergoing RARC had a mean BMI of 27.1 kg/m^2^, mean age-adjusted CCI of 7.1, and mean ASAs score of 3.2. No statistically significant differences were observed according to the preoperative features (Table 1).

The intraoperative parameters are shown in Table 2. The mean operative time was 232.8 min (SD 65.8) for ORC vs. 189.2 min (SD 42.4) for RARC (*p* = 0.04). Ureterocutaneostomy was performed in 82 patients (80.4%) after ORC and 86 (78.2%) after RARC (*p* = 0.11). Intracorporeal ureteroileocutaneostomy was performed in 20 patients (19.6%) after ORC and 24 (21.8%) after RARC (*p* = 0.13). Mean estimated blood loss (EBL) was 832.8 mL in Group A vs. 523.7 mL in Group B (*p* = 0.04). An intraoperative transfusion was performed in 32 cases (31.4%) during ORC vs. 11 cases (10.0%) during RARC (*p* = 0.03). Intraoperative complications occurred in 12 (11.8%) vs. 8 (7.3%) patients during ORC vs. RARC (*p* = 0.11). Overall, no differences were observed in the specific intraoperative complications, as shown in Table 2.

The mean length of stay was 12.6 days (SD 8.5) after ORC vs. 7.2 days (SD 4.4) after RARC (*p* = 0.02). At discharge, a mean hemoglobin (Hb) reduction of 1.8 g/dL after both ORC and RARC compared to the preoperative value was observed. Similarly, no differences were observed in preoperative and at-discharge creatinine (Cr) (Table 2). Postoperative transfusions were performed in 36 cases (35.3%) in Group A vs. 13 cases (11.8%) in Group B (*p* = 0.03). Postoperative complications occurred in 37 (36.3%) vs. 29 (26.4%) patients in Groups A vs. B, respectively (*p* = 0.05). Among these, a significant difference was observed in the rate of postoperative fever (24.5% vs. 10.9%, *p* = 0.03), anemization (35.3% vs. 11.8%, *p* = 0.02), acute limb ischemia (0 cases after ORC vs. 1 case after RARC, *p* = 0.04), and bowel occlusion (8 cases after ORC vs. 0 cases after RARC, *p* = 0.01) (Table 2).

Pathological characteristics are reported in Table 3. In all cases, the pathological report confirmed the pT4a stage. No significant differences were observed according to tumor histotype and lymph node invasion. Positive surgical margins were detected in 22 patients (21.6%) after ORC vs. 12 patients (10.9%) after RARC (*p* = 0.05). The rate of neoadjuvant chemotherapy (CT) and adjuvant CT or radiotherapy (RT) was comparable between the two groups. Median follow-up was 72.3 months in Group A vs. 65.9 months in Group B (*p* = 0.06). No differences were recorded according to the patients’ status at last follow-up, as shown in Table 3. Overall survival (OS) rates were 53.2% vs. 55.3% at 24-month (*p* = 0.13) and 35.7% vs. 37.8% at 60-month (*p* = 0.14) for ORC vs. RARC. Cancer-specific survival (CSS) rates were 47.6% vs. 48.7% at 24-month (*p* = 0.08) and 27.1% vs. 29.3% at 60-month (*p* = 0.10) for ORC vs. RARC, whereas the disease-free survival (DFS) rates were 33.4% vs. 35.6% at 24-month (*p* = 0.12) and 23.3% vs. 26.8% at 60-month (*p* = 0.09) for ORC vs. RARC, respectively.

## 4. Discussion

Due to the features of the procedure itself and the often weak general health status of the patients, radical cystectomy is related to a significant incidence of complications. During the last decades, robot-assisted surgery has spread in bladder cancer treatment in order to take advantage of the benefits of minimally invasive surgery. In adjunct, the Enhanced Recovery After Surgery (ERAS) program aims to enhance patient recovery and discharge by optimizing preoperative, intraoperative, and postoperative management through a multidisciplinary team involving urologists, anesthesiologists, and nurses [31]. However, the treatment of patients with locally advanced disease remains a challenge. In an analysis of the National Cancer Database comparing robotic versus open radical cystectomy among locally advanced and node-positive patients, the robotic approach was associated with superior unadjusted survival and lower unadjusted 30- and 90-day mortality, lower positive margin status, and shorter length of stay. However, after adjusting for confounding covariates, no differences were found, except for shorter hospitalization [32]. Volz et al. showed that multiple locally advanced lesions and the female gender were independent predictors of worse survival outcomes in pT4 patients undergoing surgery [33].

Different strategies have been developed to avoid surgery or to improve surgical outcomes, including multimodal therapy or neoadjuvant chemotherapy. However, these strategies rarely guarantee the control of the disease, and when a salvage cystectomy becomes necessary due to life-threating complications, the procedure is associated with significantly higher morbidity and worse outcomes. In patients with locally advanced disease, the survival benefit of neoadjuvant chemotherapy may be questionable. Although downstaging possibly improving surgical eradication has been described, the response to neoadjuvant chemotherapy is not obvious, and subsequent surgery may be significantly interfered by tumor growth [34]. Multimodal bladder-preserving therapy has been shown to be a reliable option for selected patients with localized disease [35,36]. On the contrary, it can lead to local complications negatively impacting on quality of life in non-selected patients [37,38]. Indeed, a complete endoscopic resection of the primary tumor is not possible when the disease grows beyond the bladder wall as in T4 stage, and response to radio-chemotherapy is improbable in these cases [39,40]. In a study of 21 patients with T4 bladder cancer who were subjected to primary radical cystectomy, 11 patients were still alive after a follow-up period of 20 months (7 patients with pT4a and 4 patients with pT4b tumors). During follow-up, 10 patients died of progressive disease within a mean time of seven months postoperatively. Complete eradication of the tumor was not possible in one case, and positive surgical margins were detected in another three patients. Only one patient died due to a complication (enterocutaneous fistula), while the others died of progressive disease during follow-up. The authors concluded that primary cystectomy for the treatment of T4 bladder cancer is feasible and associated with acceptable morbidity and mortality. Moreover, the prevention of complications which significantly worsen quality of life is an additional advantage [34].

In a review on the current knowledge of RARC, oncological outcomes were found to be comparable to ORC. As for perioperative outcomes, less intraoperative bleeding, a lower intraoperative transfusion rate, a shorter length of stay, less Clavien–Dindo grade III-V complications, and a lower rehospitalization rate were commonly observed after RARC compared to ORC [41]. Our study adds evidence to the feasibility of robotic surgery in the case of pT4a bladder cancer and the possibility of benefitting from the advantages of minimally invasive surgery also in advanced disease.

Operative time was significantly shorter with RARC (Table 2). Many factors may contribute to this finding. ORC implies a certain amount of time to gain access to the pelvic cavity at the beginning of the surgery and to properly close all the layers of the abdominal wall at the end. Certainly, time to set up and to dock the robot must be taken into account as well. However, due to the complexity of all its steps, RARC is usually performed in high-volume robotic centers where a coordinated work of surgeons, nurses, and anesthesiologists in the operating room significantly minimizes the time needed to prepare the robot [42]. Moreover, high-quality intraoperative vision, magnification, and precision of movements may be other factors contributing to the shorter operative time. Notably, a mean decrease of 43.6 min in operative time may end up being the possibility to add at least one extra surgery in the scheduled operative program.

The previously mentioned advantages of robotic surgery, high-quality 3D vision, magnification, precision, and a wide range of available movements, may allow for the better control of bleeding and, therefore, could explain the significantly smaller amount of EBL, with a mean reduction of 309.1 mL with robotics compared to the open approach (Table 2). Pneumoperitoneum needed with the robotic approach further enhances bleeding control. This aspect has some positive implications. Firstly, the less time needed to perfect hemostasis, the less the total operative time is, which further explains the previously discussed result. Secondly, a significantly lower rate of intraoperative and postoperative transfusions is observed. Thirdly, we found a significantly shorter length of stay. Our results substantially confirm what is already known about the advantages of robot-assisted surgery in terms of bleeding control [43].

Interestingly, the same advantages of robotics seemed not to have an influence on the rate of intraoperative complications, which was comparable between ORC and RARC (Table 2). These data could be explained by the fact that, despite better intraoperative vision and more precise movements, the risk of vascular or visceral lesions is mainly conditioned by the stage of the disease, especially in expert hands. Indeed, a locally advanced disease may alter the quality of the surrounding tissues, creating tenacious adherences between contiguous structures and significantly complicating dissection no matter the approach used.

Differently from intraoperative complications, a lower postoperative complication rate was observed in Group B compared to Group A. The majority of patients had a low-grade complication, mainly fever treated with antibiotics or anemization requiring transfusions. One patient had acute ischemia of the lower limb following an intraoperative lesion of the right external iliac artery which required an end-to-end anastomosis. Interestingly, eight cases of bowel occlusion occurred after ORC, probably due to the extensive bowel mobilization (Table 2). Again, our results confirm previous evidence [43].

Length of stay was significantly shorter after RARC (Table 2). This result is in line with others from the literature [43], and it is the consequence of the previously mentioned advantages of minimally invasive treatment, namely a lower transfusion rate and lower postoperative complications rate, together with known faster recovery compared to open surgery.

Among pathological features, the rate of positive surgical margins was the only statistically significant difference observed in favor of RARC (Table 3). Again, this could be explained as a consequence of the already highlighted benefits of robotics, specifically high-quality 3D vision, magnification, and the precision of movements. Despite this, the oncological outcomes were not different between the two groups, probably due to the major impact of tumor stage on patients’ prognosis.

Despite its strengths, the limitations of our study need to be taken into account. Firstly, the relatively low number of patients. Secondly, the retrospective nature of the study. Thirdly, the lack of a pure-laparoscopic group to perform a more complete comparison between the available approaches. Fourthly, the lack of a centralized revision of pathological reports. Lastly, a standardized homogeneous follow-up protocol was not applied among all the centers.

## 5. Conclusions

Open and robot-assisted radical cystectomy are both feasible treatments for the management of pT4a bladder tumors. Minimally invasive surgery provides some benefits, including shorter operative time, less bleeding, a lower transfusion rate, fewer postoperative complications, and shorter length of stay. A lower rate of positive surgical margins was observed with robotics, potentially due to better intraoperative vision and higher precision of movements. However, no significant differences between the two approaches were observed in oncological outcomes, which seem to be mainly influenced by the very-high-risk nature of the disease.

## Figures and Tables

**Table 1:** Descriptive characteristics of 212 patients with pT4a bladder cancer treated with open (102 patients) vs. robot-assisted (110 patients) radical cystectomy.

Variables		Group A ORC (*n* = 102)	Group B RARC (*n* = 110)	*p*
Age, years	Mean (SD)	78.9 (10.1)	79.2 (9.2)	0.19
Gender, *n*	M/F	66/39	70/40	0.14
BMI, kg/m^2^	Mean (SD)	25.8 (6.3)	27.1 (5.2)	0.27
Age-adjusted CCI	Mean (SD)	6.9 (2.6)	7.1 (2.5)	0.25
ASAs score	Mean (SD)	2.9 (1.1)	3.2 (0.8)	0.21

ORC = open radical cystectomy, RARC = robot-assisted radical cystectomy, SD = standard deviation, M = male, F = female, BMI = body mass index, CCI = Charlson Comorbidity Index, ASAs = American Association of Anesthesiologists.

**Table 2:** Intraoperative and postoperative characteristics of 212 patients with pT4a bladder cancer treated with open (102 patients) vs. robot-assisted (110 patients) radical cystectomy.

Variables		Group A ORC (*n* = 102)	Group B RARC (*n* = 110)	*p*
Operative time, min	Mean (SD)	232.8 (65.8)	189.2 (42.4)	0.04
EBL, mL	Mean (SD)	832.8 (599.2)	523.7 (312.3)	0.04
Urinary diversion Ureterocutaneostomy Ureteroileocutaneostomy	*n* (%)	82 (80.4) 20 (19.6)	86 (78.2) 24 (21.8)	0.11 0.13
Intraoperative transfusions	*n* (%)	32 (31.4)	11 (10.0)	0.03
Intraoperative complications	*n* (%)	12 (11.8)	8 (7.3)	0.11
Type of intraoperative complication Vascular lesion Rectal Injury Bowel perforation	*n* (%)	4 (3.9) 4 (3.9) 4 (3.9)	2 (1.8) 4 (3.6) 2 (1.8)	0.09 0.23 0.09
Preoperative Cr, mg/dL	Mean (SD)	1.8 (1.3)	1.9 (1.4)	0.12
Preoperative Hb, g/dL	Mean (SD)	11.2 (2.5)	11.1 (1.9)	0.08
Discharge Cr, mg/dL	Mean (SD)	1.6 (1.1)	1.4 (0.9)	0.08
Discharge Hb, g/dL	Mean (SD)	9.4 (1.8)	9.3 (1.6)	0.12
Postoperative transfusions	*n* (%)	36 (35.3)	13 (11.8)	0.03
Postoperative complications	*n* (%)	37 (36.3)	29 (26.4)	0.05
Type of postoperative complication Fever Anemization Hydronephrosis Acute limb ischemia Bowel occlusion	*n* (%)	25 (24.5) 36 (35.3) 12 (11.8) 0 (0) 8 (7.8)	12 (10.9) 13 (11.8) 7 (6.4) 1 (0.9) 0 (0)	0.03 0.02 0.06 0.04 0.01
Length of stay, days	Mean (SD)	12.6 (8.5)	7.2 (4.4)	0.02

ORC = open radical cystectomy, RARC = robot-assisted radical cystectomy, SD = standard deviation, EBL = estimated blood loss, Cr = creatinine, Hb = hemoglobin.

**Table 3:** Pathological characteristics and follow-up of 212 patients with pT4a bladder cancer treated with open (102 patients) vs. robot-assisted (110 patients) radical cystectomy.

Variables		Group A ORC (*n* = 102)	Group B RARC (*n* = 110)	*p*
Histotype Urothelial cancer Adenocarcinoma Squamous cell Sarcomatoid	*n* (%)	63 (61.8) 2 (1.9) 28 (27.5) 9 (8.8)	70 (63.6) 3 (2.7) 27 (24.6) 10 (9.1)	0.13 0.20 0.21 0.19
Lymph node invasion	*n* (%)	55 (53.9)	57 (51.8)	0.18
pN pN1 pN2	*n* (%)	40 (39.2) 15 (14.7)	45 (40.9) 12 (10.9)	0.16 0.14
Positive margins Prostate base Mid-prostate	*n* (%)	22 (21.6) 17 (16.7) 5 (4.9)	12 (10.9) 9 (8.2) 3 (2.7)	0.05 0.04 0.10
Neoadjuvant CT	*n* (%)	8 (7.8)	12 (10.9)	0.07
Adjuvant CT Gemcitabine + Cisplatin Gemcitabine + Carboplatin	*n* (%)	24 (23.5) 9 (8.8) 15 (14.7)	24 (21.8) 11 (10.0) 13 (11.8)	0.09
Adjuvant RT	*n* (%)	5 (4.9)	6 (5.5)	0.14
Follow-up, months	Mean (SD)	72.3 (23.9)	65.9 (17.4)	0.06
90-day mortality	*n* (%)	11 (10.8)	9 (8.2)	0.12
Status at last follow-up Alive without disease Alive with disease Death for cancer Death for other cause	*n* (%)	26 (25.5) 14 (13.7) 52 (51.0) 10 (9.8)	30 (27.3) 19 (17.3) 49 (44.5) 12 (10.9)	0.19 0.10 0.21 0.24

ORC = open radical cystectomy, RARC = robot-assisted radical cystectomy, CT = chemotherapy, RT = radiotherapy, SD = standard deviation.

## Data Availability

The datasets presented in this article are not readily available due to technical and time limitations.
